# Improving diabetic and hypertensive retinopathy with a medical food containing L-methylfolate: a preliminary report

**DOI:** 10.1186/s40662-019-0147-0

**Published:** 2019-07-22

**Authors:** Jianhua Wang, Craig Brown, Ce Shi, Justin Townsend, Giovana Rosa Gameiro, Peng Wang, Hong Jiang

**Affiliations:** 1 Bascom Palmer Eye Institute, University of Miami, Miller School of Medicine, 1638 NW 10th Avenue, McKnight Building - Room 202A, Miami, FL 33136 USA; 2 Department of Ophthalmology, College of Medicine, University of Arkansas for Medical Sciences, Fayetteville, AR USA; 3 School of Ophthalmology and Optometry, Wenzhou Medical University, Wenzhou, Zhejiang China; 4 Department of Ophthalmology, Shanghai General Hospital, Shanghai Jiaotong University School of Medicine, Shanghai, China

**Keywords:** Diabetes, Homocysteine, L-methylfolate, Microaneurysms, MTHFR C677T, Retinopathy, Vitamin D

## Abstract

**Background:**

Homocysteine and vitamin D may play a role in the development of diabetic and hypertensive retinopathy in patients with diabetes mellitus (DM) and hypertension. Supplementing food with L-methylfolate and vitamin D theoretically may improve diabetic and hypertensive retinopathy, however, the outcome of these nutritional approaches has not been fully examined. A retrospective case review was done of cases of retinopathy reversal in patients on Ocufolin™ and a similar nonprescription multivitamin, Eyefolate™. In this study, they were administered L-methylfolate (2.7 mg and 3.0 mg, respectively) and vitamin D3 (4500 IU each). These dosages are significantly above the RDA but well below levels associated with toxicity.

**Case presentation:**

Seven patients had nonproliferative diabetic retinopathy (NPDR) and some of them had hypertension. One patient had only hypertensive retinopathy. All patients were instructed to take Ocufolin™ medical food as a food supplement. Baseline genetic testing for MTHFR polymorphisms was conducted. Fundus photography was used to document the fundus condition of the enrolled eyes in 8 NPDR patients at the initial and follow-up visits. Microaneurysms (MA) and exudates were observed to be improved in some trial patients. All subjects had one or more MTHFR polymorphisms. All had diabetic retinopathy, hypertensive retinopathy, or both. MAs were resolved, and exudates were decreased in 8/8 cases after taking the medical food. Retinal edema was found in 2/8 cases and improved or resolved in both cases after taking the medical food or the supplement. The best corrected visual activity was stable or improved in 8/8 cases.

**Conclusion:**

We report a series of diabetic and hypertensive retinopathy cases with MTHFR polymorphisms and the improvement of retinal microvasculature (mainly MAs) in serial fundus photography after taking a medical food or supplement containing L-methylfolate and vitamin D. It appears that the use of nutritional supplements and medical foods containing L-methylfolate and vitamin D may be effective in facilitating the improvement of diabetic and hypertensive retinopathy.

## Background

As one of the major complications of diabetes mellitus (DM), diabetic retinopathy (DR) is the leading cause of new cases of blindness among working-age adults [1, 2]. Diabetes and diabetic retinopathy are rapidly becoming more serious and are increasingly prevalent healthcare issues. Hypertension is a major risk factor for DR. Worldwide, there are more than 94 million people with DR [3]. Studies suggest that in the US, there are over 7.6 million diabetics with DR and another 6.6 million pre-diabetics with DR. Combined, there are 14,000,000 people with DR which, if untreated, can progress to visual disability [4, 5].

DR is commonly described as a microvascular disease whose drivers include increased renin-angiotensin systemic dysfunction, increased vascular inflammatory response, deregulation of growth factors, and increased vascular permeability [6]. The current standard of care treatments is the injection of anti-VEGF drugs and the use of laser photocoagulation to slow vision loss and occasionally improve vision. However, these treatments are costly, invasive, stressful, and time-consuming for both doctors and patients. Both are often necessary for more advanced DR, which already pose a threat to vision but are generally avoided in the earlier stages of the disease. Hence, beyond good control of blood sugar and blood pressure, less invasive complementary treatments are needed to slow the progression of early DR. Ideally, such complementary treatments would not interfere with the current standard of care treatments. Previous studies found increased homocysteine associated with diabetic retinopathy [7, 8]. The homocysteine affects vascular endothelial growth factor in the retina [9, 10], which is also associated with the regulation of angiogenesis [11]. Reducing homocysteine is one such approach that addresses another driver of DR, without impairing anti-VEGF and laser therapies.

The methylenetetrahydrofolate reductase gene (MTHFR) encodes for methylenetetrahydrofolate reductase, an enzyme involved in the methylation of B12 and homocysteine [12–15]. The MTHFR polymorphisms impair enzymatic activity, resulting in decreased serum L-methylfolate, serum methylcobalamin, and elevated serum homocysteine levels. These have been shown to cause hypertension [12–14], small vessel disease, and DR [16]. The MTHFR polymorphisms thus contribute to diabetic and hypertensive retinal vasculopathy [12–14, 17–20].

Vitamin D is a key regulator of several important metabolic functions [21, 22]. Vitamin D insufficiency is associated with increased CRP, arterial stiffness, and endothelial dysfunction [22]. Vitamin D deficiency increases levels of parathyroid hormone (PTH), which increases insulin resistance and is associated with an increased incidence of diabetes [23]. Finally, vitamin D deficiency in type 2 diabetes patients increases the risk of DR [24]. The deficiency of vitamin D may play an important role in the progression and severity of DR [25–27].

Treating diabetic and hypertensive retinopathy with vitamin D and L-methylfolate may be helpful; however, the efficacy of these treatments has not been fully studied. Food supplementation is safe, simple, and inexpensive. A previous study reported that folic acid therapy reduces the risk of stroke [28]. Folic acid supplementation was also found to be associated with reduced risk of retinal microangiopathy in adults with hypertension complicated with DM [17]. Another study reported that the medical food Ocufolin™ may have a marked improvement in retinal artery occlusion outcome as late as 24 h post occlusion [29]. L-methylfolate (5-MTHF) more readily crosses the blood brain barrier and blood retinal barrier than folic acid and has less toxicity than folic acid, thus making it a more potent choice for retinal therapy than folic acid [30–32]. Here, we present the findings of 8 patients with diabetic and/or hypertensive retinopathy who took food supplements with supratherapeutic levels of L-methylfolate and vitamin D.

## Case presentation

### Methods

During a prospective study of the effects of medical food, Ocufolin™ on retinal blood flow in patients with diabetic retinopathy, visible improvements of baseline retinopathy were noted. This prompted a retrospective review of other patients treated with Ocufolin™ and a similar product, Eyefolate™ for visible improvements of retinopathy.

We reviewed 7 retrospectively identified cases from the Eye Center, Fayetteville, AR and 1 case from Bascom Palmer Eye Institute, University of Miami, Miami, FL. This study was performed from Oct 2013 to Dec 2018. The Washington Regional Institutional Review Board waived review of this retrospective case study of anonymized case data from the Fayetteville site, and the University of Miami Institutional Review Board approved the prospective study conducted at the University of Miami. Written informed consent to be included in this study was obtained from each study subject.

All subjects had been examined by experienced ophthalmologists. Every patient received a full ophthalmic examination including slit-lamp biomicroscopy, intraocular pressure (IOP) measurement, refraction, best corrected visual acuity (BCVA) and fundus indirect ophthalmoscopic examination, and fundus photography. Baseline diabetic retinopathy severity scores were determined by the retinal specialist, according to the International Clinical Diabetic Retinopathy Disease Severity Scale. Genetic testing was performed by MyGenetx Laboratory, LLC (Franklin, TN) or Quest Diagnostics. In addition, other blood tests were performed by Quest Diagnostics, including homocysteine, vitamin D, vitamin B12 and HbA1c. Standard fundus photos were taken using a Zeiss Visucam NM/FA at the Fayetteville Eye Center. A Topcon (50DX, Topcon Medical Systems, Inc., Oakland, NJ) fundus camera and a Zeiss Clarus fundus camera (Model 500, Carl Zeiss Meditec, Inc., Dublin, CA) were used at the Bascom Palmer Eye Institute.

Case 8 was one of the patients recruited in a prospective clinical study using Ocufolin™ medical food supplementation at the Bascom Palmer Eye Institute. This patient was imaged using the retinal function imager (RFI), an advanced ophthalmic imaging modality based on a fundus camera. The retinal blood flow velocity was measured. The detailed information and applications of RFI were reviewed previously [33]. Before the study, the pupil of this patient was dilated with 1% tropicamide. A field of view of 4.3 × 4.3 mm^2^ (20 degrees) was used. The retinal blood flow velocity was measured in the arterioles and venules.

### Results

Basic information from the 8 cases is presented in Table 1. All subjects were Caucasians and diagnosed with nonproliferative diabetic retinopathy (NPDR) and/or hypertensive retinopathy (HR). Three were females and five were males.

**Table 1:** Basic information, gene and medical food of the current series of subjects

Subject	Age (years)	Race	Gender	Present DR stage	Gene type	Medical Food	Medical Food treatment duration (years)	Other ophthalmic complications
1	62	Caucasian	Female	NPDR OU	MTHFR C677T/A1298C	Eyefolate	4	Cataract OU
2	82	Caucasian	Female	NPDR OU	MTHFR C677T/A1298C	Eyefolate	4	Dry Macular Degeneration
3	84	Caucasian	Female	NPDR OU	MTHFR C677TT Homozygous	Eyefolate	5	Low Tension Glaucoma, Branch retinal vein occlusion OS
4	59	Caucasian	Male	NPDR OU	MTHFR C677TT Homozygous	Eyefolate & Ocufolin	3	Glaucoma suspect, Peripheral Neuropathy, Cataract
5	76	Caucasian	Male	non diabetic	MTHFR C677T/A1298C	Eyefolate	4	Hypertension, small BRVO, Age-related Macular degeneration OU, cataract OU
6	56	Caucasian	Male	NPDR OU	MTHFR A1298CC Homozygous	Eyefolate & Ocufolin	3	Vitreous Hemorrhage OS, Vitrectomy OU
7	64	Caucasian	Male	NPDR OU	None	Eyefolate	1	None
8	68	Caucasian	Male	NPDR OS	None	Ocufolin	0.5	Posterior vitreous detachment OU

*NPDR* non-proliferative retinopathy, *HR* hypertensive retinopathy

Among them, 7 subjects took Eyefolate™ (3 subjects switched to Ocufolin™) and one subject took only Ocufolin™. Detailed information of these formulations is listed in Table 2. During the food supplement treatment period, 2 subjects had BCVA improvement and 3 subjects had mild BCVA decline (Table 3). The remaining 3 subjects remained at the same BCVA. All subjects showed resolution of intra-retinal hemorrhages, microaneurysms (MA), and or the reduction of exudates on the fundus photograph. Detailed information of each case is presented below.

**Table 2:** Ingredients of two food supplements mentioned in this study (per capsule). Dose: 3 capsules with the breakfast meal

Ingredient (each capsule contains)	Eyefolate™	Ocufolin™
Metafolin^R^ L methylfolate	1000 mcg	900 mcg
Vitamin C	45 mg	45 mg
Vitamin D	1500 IU	1500 IU
Vitamin E (natural tocopherols)	−0−	7.5 IU
Vitamin B1	1 mg	1.5 mg
Vitamin B2 Vitamin B3	10 mg 15 mg	10 mg −0−
Vitamin B6 (P-5-P) Vitamin B7 (Biotin)	2 mg 100 mcg	3 mg −0−
Vitamin B12 (methylcobalamin)	500 mcg	500 mcg
Calcium-D-Pantothenate (Vitamin B5)	5 mg	5 mg
Zinc Oxide	25 mg	26.75 mg
L-Selenomethionine	20 mcg	20 mcg
Cupric Oxide	0.667 mg	0.667 mg
N-Acetyl Cysteine	−0−	180 mg
Alpha Lipoic Acid	180 mg	−0−
Lutein	3.35 mg	3.35 mg
Zeaxanthin	250 mcg	700 mcg
Astaxanthin	50 mcg	−0−

**Table 3:** The BCVA and fundus photo findings before and after taking medical food

Subject	Medical Food	Medical food treatment duration (years)	BCVA (before)	BCVA (after)	Fundus Photo findings (before)	Fundus Photo Findings (after)
1	Eyefolate	4	20/40 OU	20/30 OD, 20/25 OS	Mild dot and blot retinal hemorrhages, exudates, CME	Fewer MAs, fewer hemorrhages, less CME
2	Eyeflolate	4	HM OD, 20/20 OS	HM OD, 20/25 OS	Intra-retinal hemorrhages and 2 small MAs	Only 1 faint MA left
3	Eyeflolate	5	20/150 OD, 20/25 OS	20/400 OD, 20/25 OS	Large MA, Exudates, mild CME	MA resolved, Exudate resolved, CME decreased
4	Eyefolate & Ocufolin	3	20/20 OU	N/A	Multiple MAs and retinal hemorrhages	MAs and retinal hemorrhages resolved
5	Eyefolate	4	20/20 OU	20/40 OD, 20/25 OS	Multiple MAs and exudates	Multiple MAs and exudates resolved on Eyefolate then recurred after he discontinued them.
6	Eyefolate & Ocufolin	3	20/30 OD, 20/400 OS	20/25 + 1 OD, 20/200 OS	Some MAs, exudates, silver wiring	MAs and exudates resolved, persistent silver wiring
7	Eyefolate	1			Intra-retinal hemorrhage	Intra-retinal hemorrhage resolved
8	Ocufolin	0.5	20/20 OU	20/20 OU	MA	MA resolved

*BCVA* best corrected visual acuity, *HM* hand motion, *MA* microaneurysms, *CME* cystic macular edema

### Case 1

**Fig. 1:**
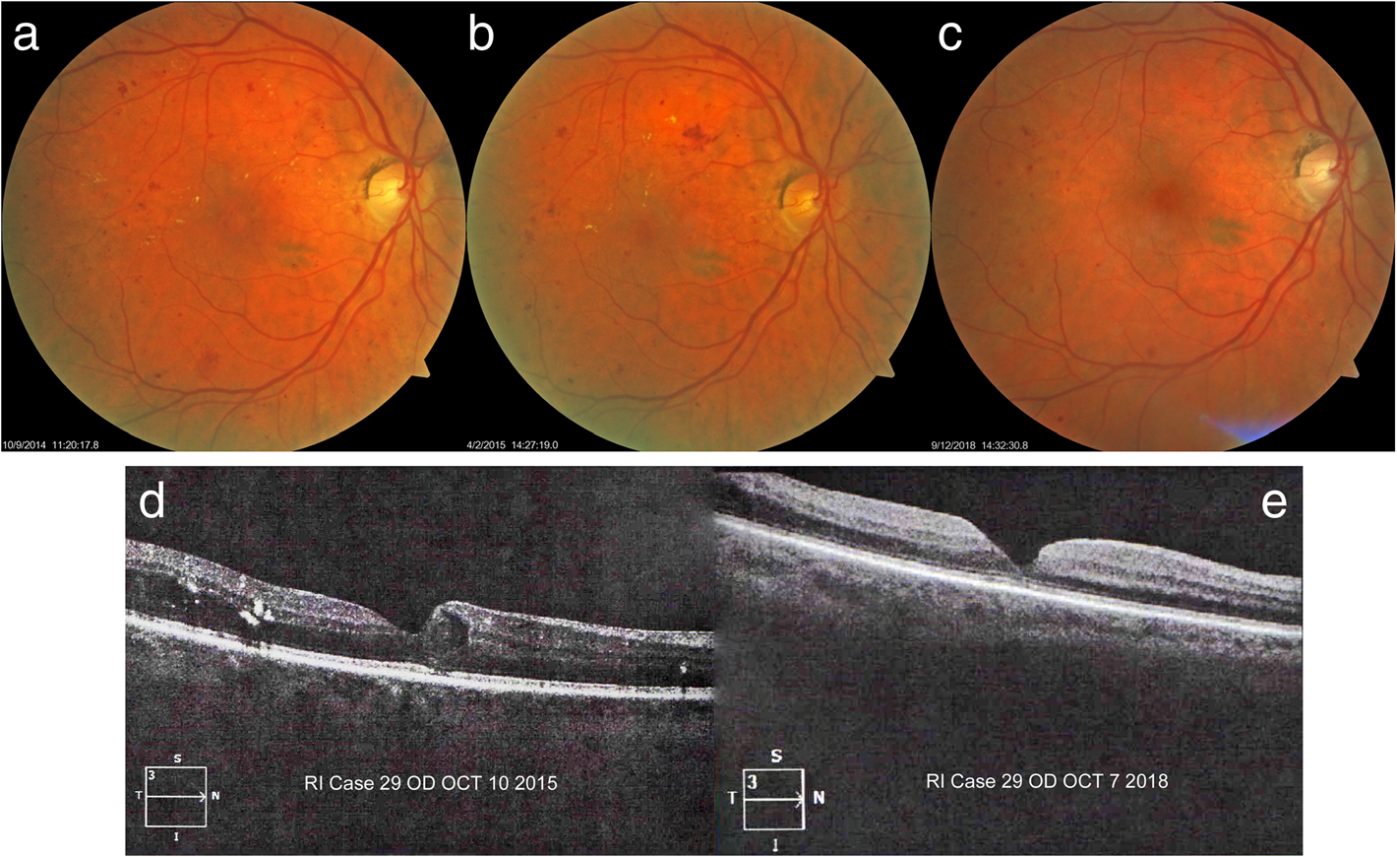
Follow-up of first case fundus photos and OCT images. The fundus photograph fewer hemorrhages and MAs after taking Eyefolate™ (**a**, **b**, **c**). The diabetic cystic macular edema resolved at a later visit (**e**) on the OCT B-scan using Zeiss Cirrus HD-OCT (Model 5000) compared to the previous visit (**d**). OCT: optical coherence tomography; MA: microaneurysms

A 67-year-old woman presented with bilateral NPDR and treated hypertension. BCVA was 20/40–2 OU at the first visit in April 2014. Fundus photos showed multiple MAs, mild dot and blot retinal hemorrhages with some exudates in the right eye (OD) (Fig. 1a). She was instructed to take 3 capsules of Eyefolate™ daily with food. In October 2015, the BCVA was 20/50 in OD, and 20/30 in the left eye (OS). Reduced MAs and retinal hemorrhages were observed (Fig. 1b). At that visit, mild diabetic cystoid macular edema (CME) was found in the OCT image (Fig. 1d). In September 2018, the subject received bilateral intraocular lens (IOL) implantation surgery after which the BCVA improved to 20/30 in OD and 20/25 in OS. The right eye showed fewer hemorrhages and MA (Fig. 1c) and less CME on OCT examination (Fig. 1e).

### Case 2

**Fig. 2:**
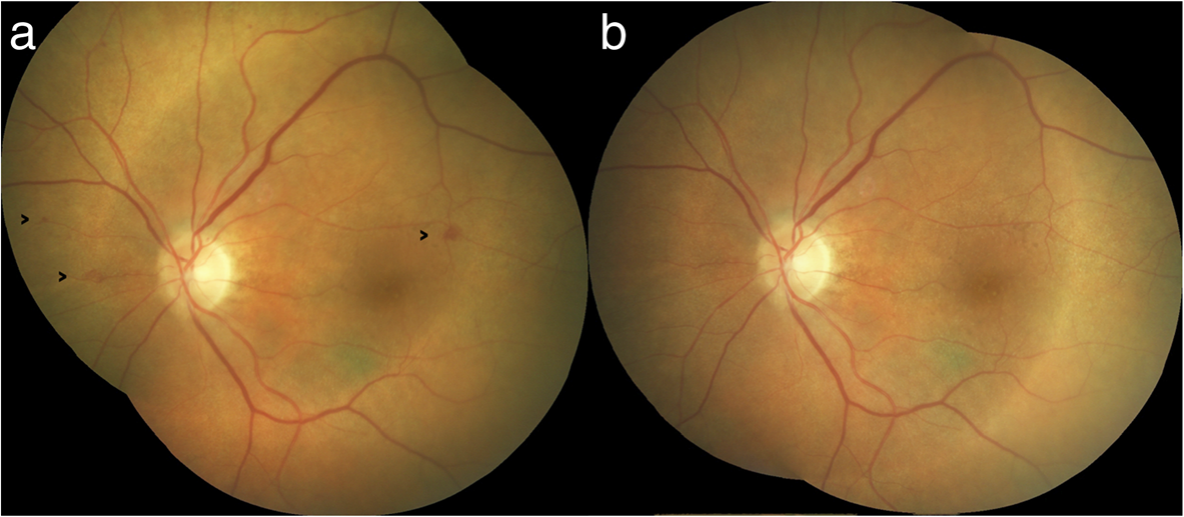
Follow-up of the second case fundus photos. Intra-retinal hemorrhages and 3 small MAs (black arrows) were seen in the fundus photo in OS eye (**a**) at the first visit. At the second visit, only 1 faint MA remained, and the hemorrhages were resolved (**b**) after taking Eyefolate™ for four months. MA: microaneurysms

An 82-year-old woman presented with diet-controlled type 2 diabetes and medication controlled hypertension. In June 2014, the BCVA in OD was hand movement (HM) due to an old macular scar and the BCVA in OS was 20/20. Intra-retinal hemorrhages and 2 small MAs were found in the fundus photo OS (Fig. 2a). She began 3 capsules of Eyefolate™ daily with breakfast. In October 2014, her BCVA remained HM in OD and 20/25 OS, but the hemorrhages resolved and only 1 faint MA was seen in the fundus photography in OS (Fig. 2b).

### Case 3

**Fig. 3:**
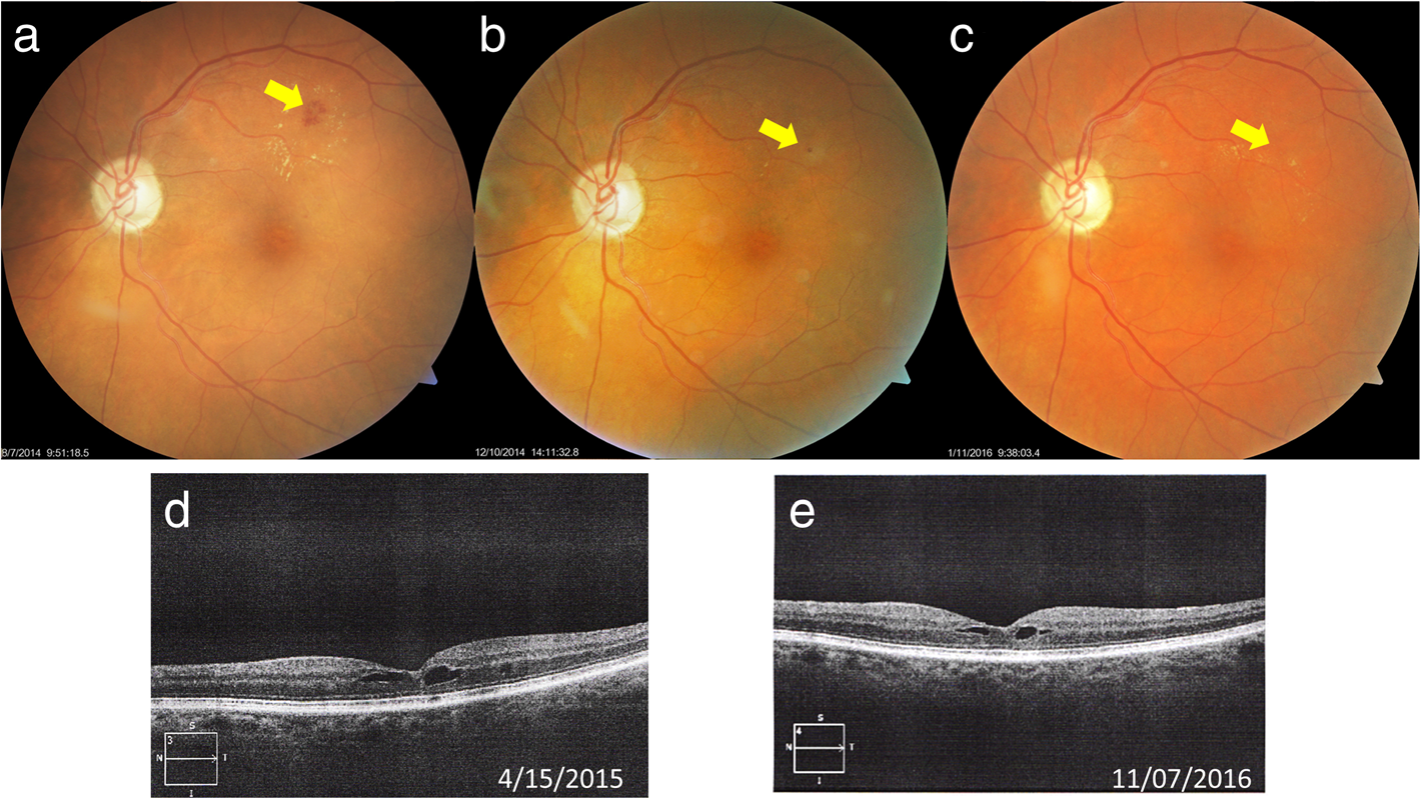
Follow-up of third case fundus photos. Some exudate leakage was found at the August 2014 visit (**a**). After taking Eyefolate™, the large MA (yellow arrow) was reduced and the exudate was smaller at the Dec 2014 visit (**b**). The MA and exudates (yellow arrow) were resolved by the January 2016 visit (**c**). In addition, at the November 16 visit (**e**), the diabetic cystic macular edema was reduced from the 2015 visit (**d**) and later visit

An 84-year-old woman presented with a seven-year history of type 2 diabetes and medically controlled hypertension. She had a history of amblyopia OD and a resolved branch retinal vein occlusion (BRVO) in the left eye. In October 2013, the BCVA was 20/150 OD and 20/25 OS. A large MA in the superior temporal region with exudates is seen in the fundus photo OS. She was instructed to take 3 capsules of Eyefolate™ daily. In August 2014, the BCVA was 20/200 OD and BCVA OS was improved to 20/20. Decreased exudation was found in OS fundus photo (Fig. 3a). In December 2014, the BCVA was 20/200 OD and 20/25 OS. The large exudates had almost completely resolved OS (Fig. 3b). In January 2016, the BCVA was 20/400 OD and 20/25 OS. The MA was resolved in the OS fundus photo (Fig. 3c) in 2016.

### Case 4

**Fig. 4:**
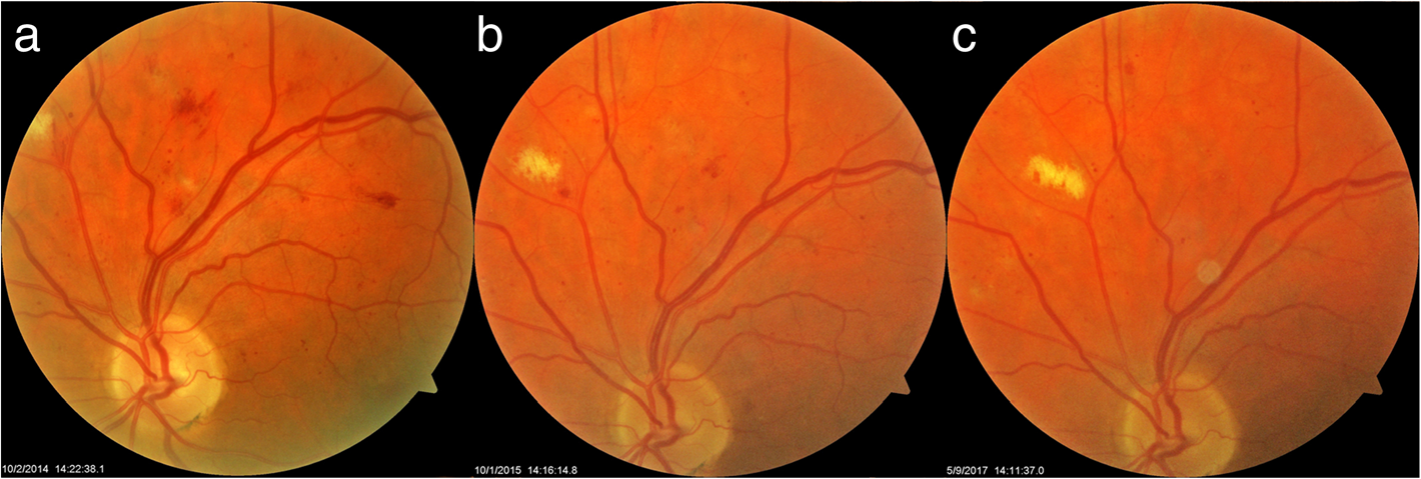
Follow-up of the fourth case fundus photos. The number of retinal hemorrhages and MAs on the fundus photo declined and exudates were resolved after taking the supplements (**a**-**c**). MA: microaneurysms

The fourth case was a 59-year-old man with an 11-year history of type 2 diabetes who had required insulin for the past 8 years. On his first visit in October 2014, the BCVA was 20/20 OU. Multiple small intra-retinal hemorrhages and MAs with exudates superotemporally were found OS as seen in the fundus image (Fig. 4a). He began 3 capsules of Eyefolate™ daily. In October 2015, the number of retinal hemorrhages and MAs declined OS (Fig. 4b), then he switched from Eyefolate™ to Ocufolin™. In April 2016, the number of hemorrhages and MAs continued to decline, and the exudates resolved OS (Fig. 4c).

### Case 5

**Fig. 5:**
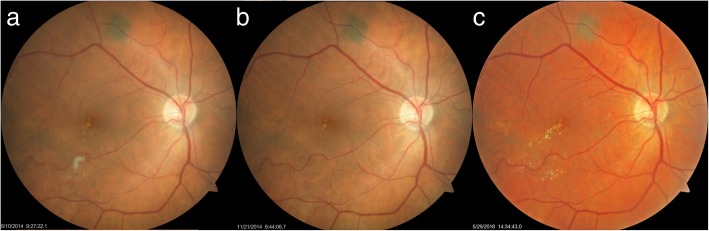
Follow-up of fifth case fundus photos. Baseline findings of drusen, exudates, a cotton wool spot and microaneurysms (**a**). MAs were smaller and the exudates and cotton wool spot resolved after taking Eyefolate™ for 5 months (**b**). MAs and exudates returned 3 ½ years after quitting Eyefolate™ (**c**). The foveal drusen did not change. MA: microaneurysms

A 76-year-old nondiabetic man was examined with a 10 year history of medication controlled hypertension and dry age-related macular degeneration (AMD) (drusen) OU. In June 2014, multiple small MAs with exudates and one cotton wool spot was found OD as seen in the fundus photo (Fig. 5a) inferotemporal to small drusen. The retinopathy was likely initiated by a small BRVO, with hypertension as a continuing co-factor, the BCVA was 20/20 OU. He began to take 3 capsules of Eyefolate™ daily. In December 2014, resolution of the cotton wool spot, exudates, and MAs was found OD (Fig. 5b). In May 2018, smaller MAs and exudates reappeared in the original locations after he had self-discontinued the Eyefolate™ (Fig. 5c).

### Case 6

**Fig. 6:**
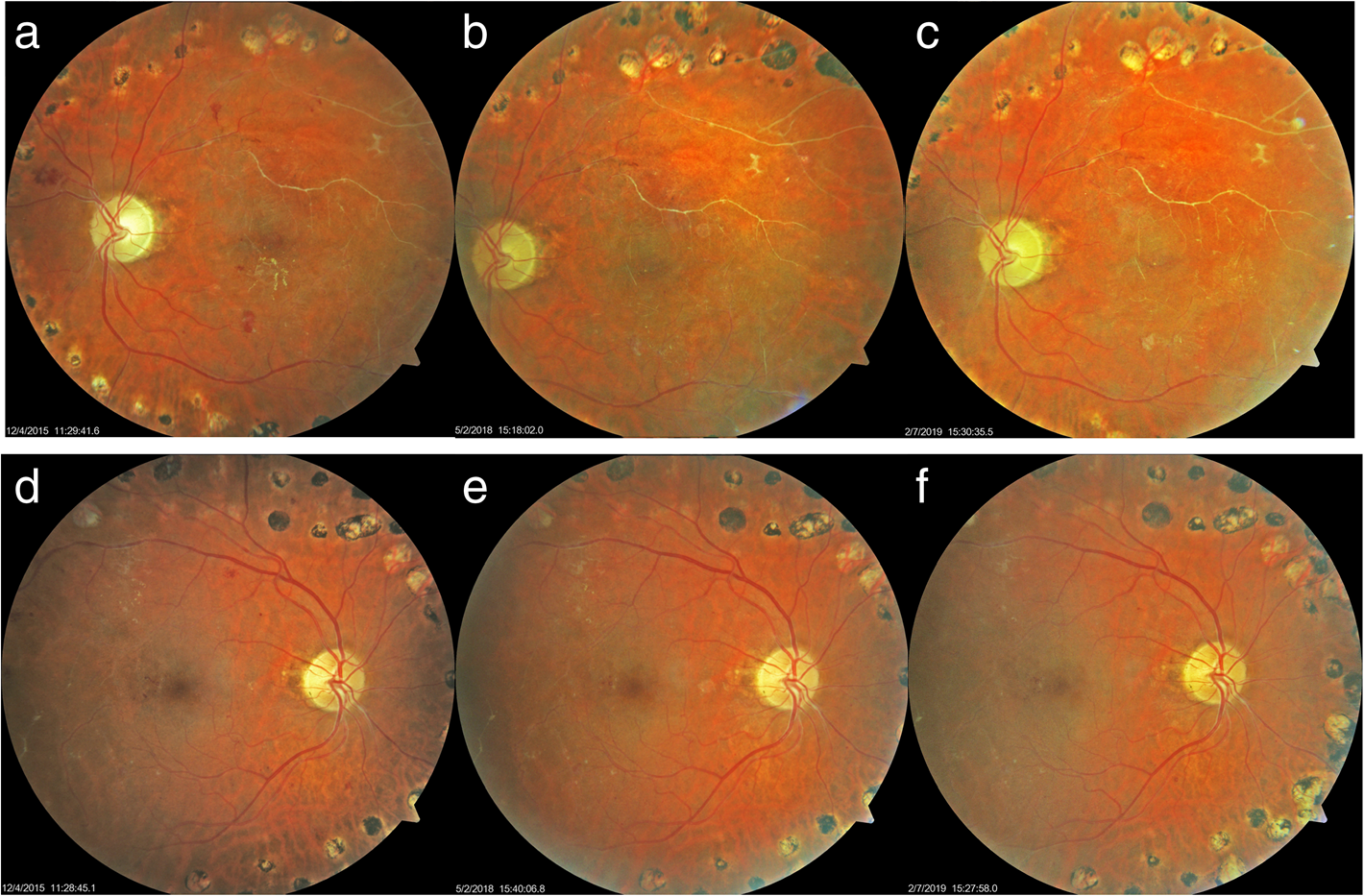
Follow-up of sixth case fundus photos. After taking Eyefolate™ then Ocufolin™, MAs, and exudates resolved. He remains free of MAs and exudates in 2/2019 (**c**). OS show resolution of intra-retinal hemorrhages, exudates, and MAs improved and remained stable over more than 3 years of follow up from 12/2015 through February 2019 (**a**-**c**). OD show resolution of intra-retinal hemorrhages and MAs between 12/2015 and 5/2018 (**d**-**f**). MA: microaneurysms

The sixth case was a 56-year-old man with a 15-year history of NPDR OU. He had a remote history of pan-retinal photocoagulation and Avastin injections for the severe proliferative disease. In June 2015, his BCVA OD was 20/30, his BCVA OS was HM. He had bilateral intra-retinal hemorrhages and MAs with old retinal scarring and silver wiring of the vessels. He began taking 3 capsules of Eyefolate™. In July 2015, his BCVA remained at 20/30 OD but improved to 20/400 OS. He switched from Eyefolate™ to Ocufolin™. In May 2016, his BCVA of this patient remained at 20/25 + 1 OD and improved to 20/200 OS. Figures 6d-f OD shows resolution of intra-retinal hemorrhages and MAs between 12/2015 and 5/2018. Figures 6a-c OS show resolution of intra-retinal hemorrhages and exudates. MAs improved then remained stable over more than 3 years of follow up from 12/2015 through February 2019.

### Case 7

**Fig. 7:**
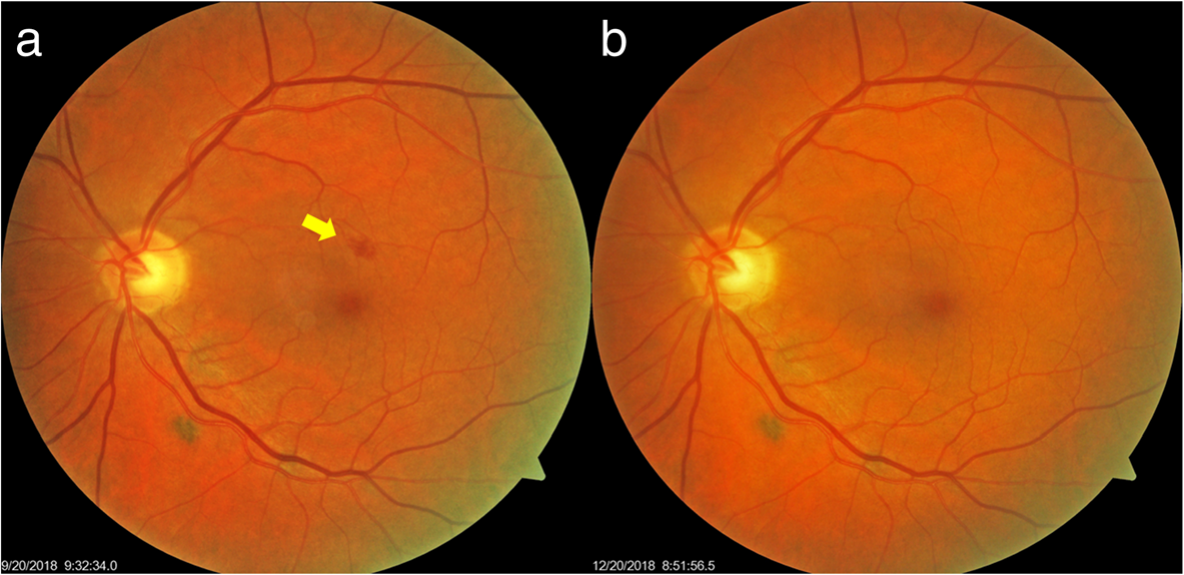
Follow-up of seventh case fundus photos. Between September 2018 (**a**) and December 2018 (**b**), the intra-retinal hemorrhage (yellow arrow) resolved after taking Eyefolate™

The seventh case was a 64-year-old man with a 10 year history of medication controlled hypertension and newly diagnosed type 2 diabetes. In August 2018, a diabetic intra-retinal hemorrhage was found OS (Fig. 7a) and he then began to take Eyefolate™. By December 2018, the intra-retinal hemorrhage was resolved (Fig. 7b).

### Case 8

**Fig. 8:**
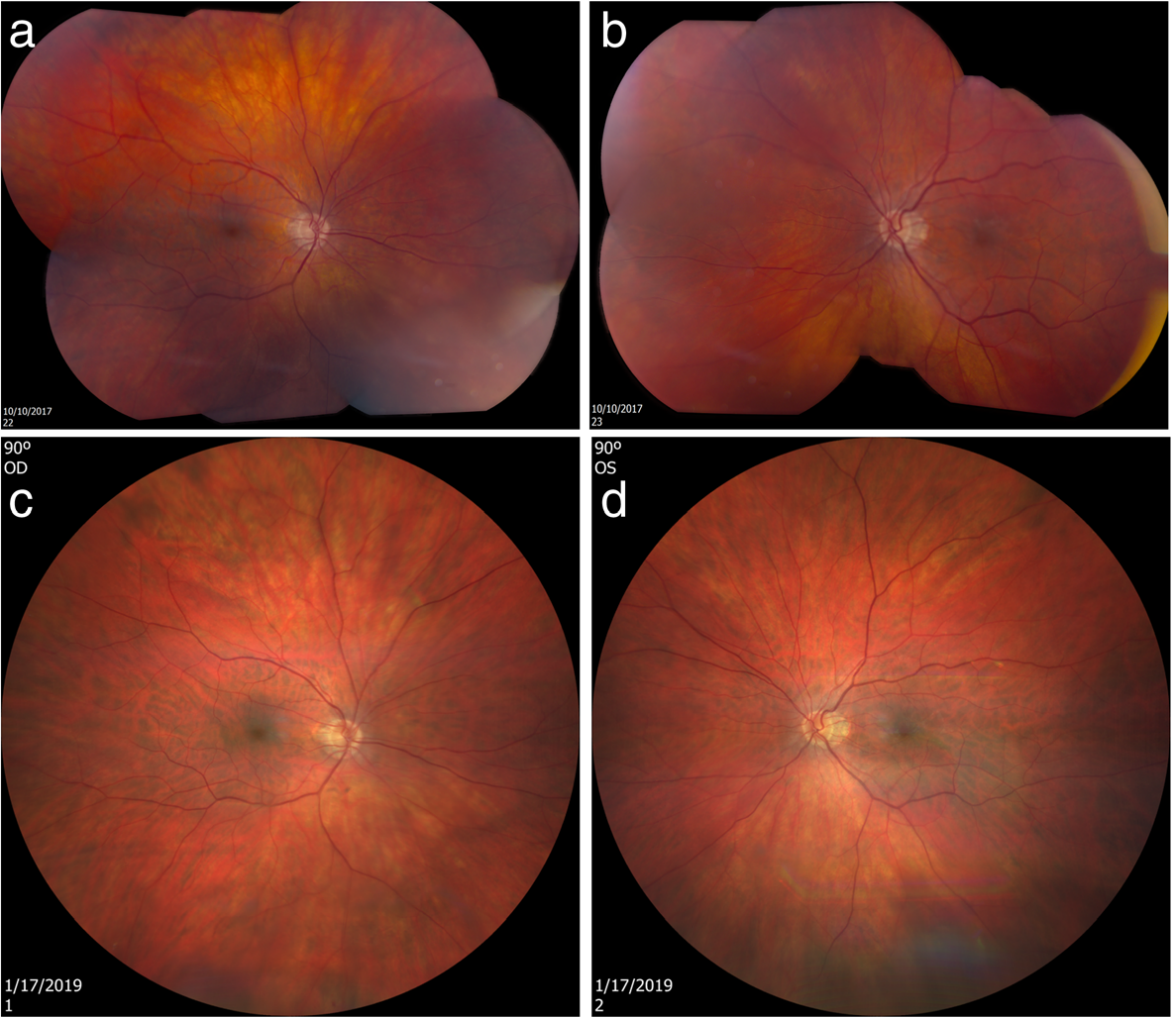
Follow-up of eighth case fundus photos. The top images were taken using Topcon DX fundus camera on October 10, 2017, before the patient took Ocufolin™ (**a**, **b**). Close inspection of fundus photos showed two MAs in the periphery of the superonasal quadrant OS. The bottom photos were taken using Zeiss Clarus 500 on January 17, 2019, after the patient participated in a clinical trial of Ocufolin™ for 6 months (**c**, **d**). The MAs in the superior nasal periphery of the OS eye resolved in the photo taken in January 2019

The eighth case was a 68-year-old man with NPDR OS. Initially, he was diagnosed with mild NPDR OS (October 2017). The BCVA was 20/20 OU. A MA was apparent in the superonasal quadrant OS in 2017 (Fig. 8). He participated in a clinical trial of Ocufolin™ at the Bascom Palmer Eye Institute, University of Miami and was instructed to take Ocufolin™ on December 19, 2017. During the April 2018 office visit, the BCVA remained 20/20 OU and the macular edema had resolved (Fig. 8). A dilated fundus examination indicated no visible retinopathy. In January 2019, a dilated fundus documented absence of macular edema and no evidence of background diabetic retinopathy (Fig. 8). Detailed inspection of the fundus photos shows two small MAs in the superior nasal periphery of the OS eyes in the fundus photo taken in October 2017 and resolved in the photo taken in January 2019. The retinal blood flow was increased at the six-month study visit (June 11, 2018, Fig. 9).

**Fig. 9:**
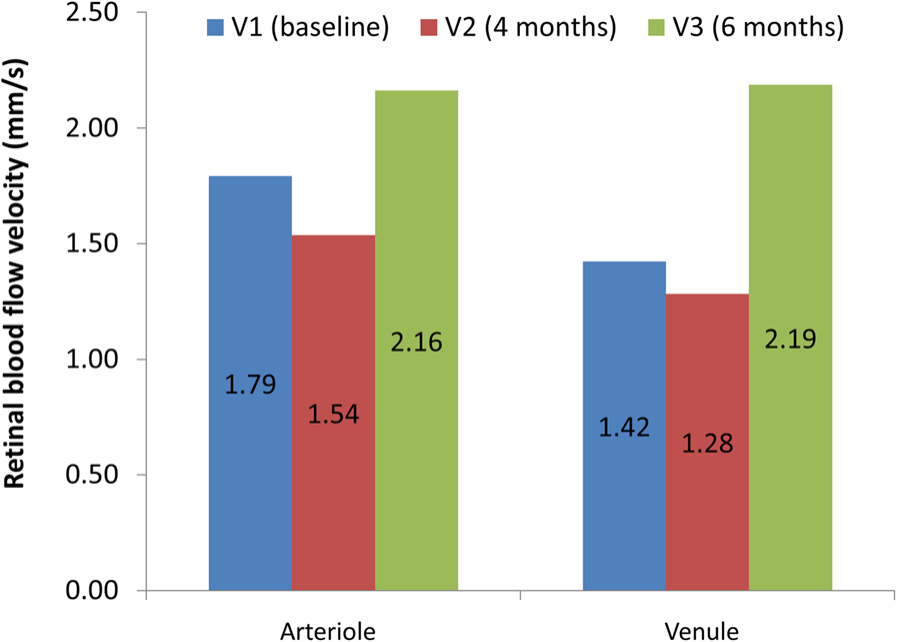
Retinal blood flow velocity of the eighth case. Retinal blood flow velocity was measured during study period of 6 months when the patient took Ocufolin™

## Discussion and conclusions

DM and hypertension cause microvascular abnormalities, including DR and hypertensive retinopathy (HR) [14, 34–37]. Microvascular abnormalities, especially retinal arterial narrowing and retinal hemorrhages, have been used to monitor the impact on the microvasculature in the body as a whole, in the central nervous system, and small vessel white matter disease [38, 39]. In addition, retinopathy poses a threat to vision and may cause blindness. Small vessel abnormalities and formation and turnover rates of MAs in the retina have been regarded as reliable predictors of the onset and progression of DR [28]. The newer MAs in successive fundus photographs, the higher probability of progression in vision-threatening DR [40].

Our study presents a case series of patients with NPDR and HR and their findings after taking a medical food containing L-methylfolate and vitamin D. The primary findings were the resolution of retinal hemorrhages and MAs, as well as the reduction of exudates and macular edema. This suggests that a supplement or medical food containing therapeutic doses of L-methylfolate and vitamin D may be a safe and non-invasive addition to our treatments of NPDR and HR.

Both Eyefolate™ and Ocufolin™ contain L-methylfolate, which targets the ischemic consequences of reduced function polymorphisms of the MTHFR gene in patients with DM. Wicken et al. reported that the C667T was present in 40% of the Caucasian American population, 50% of Europeans and a lower percentage in African-Americans and Asian-Americans [41]. MTHFR C677T and A1298C polymorphisms lead to a reduction in the methylation of tetrahydrofolate, which results in decreased available L-methylfolate, methylcobalamin (methyl B12), and the elevation of homocysteine. These further lead to elevated blood pressure damaging the small vessels [42].

In a recent study of 24 patients with DM, plasma homocysteine concentration was significantly decreased after 3 months of taking Ocufolin™ [43]. In that study, retinal blood flow measured using Doppler OCT did not reach a significant level. However, only 1 capsule of Ocufolin™ was taken during that study period, which may have been too low a dose to affect retinal blood flow [43]. In contrast, all cases reported here took 3 capsules daily. Case 8 in our study had increased retinal blood flow velocity, which was directly measured using RFI. We hypothesize that decreased homocysteine resulted in improved retinal blood flow due to the mitigation of homocysteine-induced narrowing of retinal artery caliber [44]. Since Eyefolate™ and Ocufolin™ lower homocysteine [43], their use seems to lead to an improvement in retinal blood flow, resulting in the improvement of retinopathy. The findings of our series of patients with retinopathy support the speculation, although further clinical studies are necessary.

Several vascular diseases such as ischemic cerebral stroke, small vessel disease, and retinal vein occlusion have been found to associate with MTHFR polymorphisms [28, 37, 45]. Also, MTHFR polymorphisms are commonly recognized risk factors for retinal vascular disease, including DR progression [34]. Case 5 seems to demonstrate improvement in retinal vein occlusion after the use of the medical food. Huo et al. reported that folic acid therapy could reduce the risk of the first stroke, suggesting that a similar approach may slow the progression of vascular disease [8]. Studies show that L-methylfolate is a better choice than folic acid with reduced toxicity and better penetration of the central nervous system and retina [30–32].

The improvement of DR of our patients that may also in part be attributed to the pharmacological doses of vitamin D in the present study. Vitamin D plays a role in anti-inflammatory and immunosuppressive activities and its potential inhibitory effect on angio-neogenesis has been hypothesized [24, 46, 47]. Hypovitaminosis of vitamin D increases the risk of DM [46–48]. Among DM patients, vitamin D deficiency is found to associate with a higher risk of DR [24, 46–48]. The potential relationship between vitamin D status and the DR severity is still controversial [49, 50]. Brown reported a case of branch retinal artery occlusion treated more than 24 h post occlusion with Ocufolin™ with a remarkably improved outcome [29]. Our findings support these earlier reports that the microvascular abnormalities (i.e., hemorrhages, MAs, exudates, and macular edema) were reduced after taking the products containing L-methylfolate and vitamin D.

BCVA is an important component of the visual function. Stability or improved BCVA is the desired outcome treatment of DR. However, DR is often associated with other vision damaging diseases. Case 2 showed a decreased BCVA, which may be due to concurrent AMD. In other cases, amblyopia, cataract, and preexisting vascular disease affected the visual outcomes. Encouragingly, the BCVA in the majority of cases remained stable during the study period. Further study with larger sample sizes and more strict inclusion criteria may help identify the effect of these nutrients on BCVA.

As the first report of a retrospective series of cases that shows a beneficial effect of nutritional supplementation of vitamin D and L-methylfolate on DR and HR, our study also has several limitations. It is not prospective. The lack of a quantitative tool impairs objective observation of the progression of DR and quantification of the dose effect. Further prospective studies with more advanced image modalities such as OCT angiography may provide a better understanding of the benefits to the retinal microvasculature. Second, all patients identified with this positive response were adult Caucasians. Prospective studies with larger sample sizes and community-based studies can stratify patients with racial diversity and their outcome. Third, as mentioned above, the other ophthalmic conditions of these subjects may affect the observations, due to the limited case series. Further prospective large-scale and double-blind studies are needed. Fourth, although the retinal blood flow was increased in case 8, results from a larger sample size are needed to validate the findings. Our ongoing prospective retinal blood flow trial with Ocufolin™ intervention will shed light on the mechanism of these unusual and encouraging case reports, as reported in the present study.

In conclusion, we report a series of DR and HR cases with MTHFR polymorphisms and improvement of retinal microvasculature (mainly hemorrhages, MAs, and exudates) in fundus photographs. It appeared that the use of a carefully formulated medical food which includes L-methylfolate and vitamin D may be effective in facilitating the improvement of DR. Future clinical trials are also needed to show optimal dosing for such medical foods and supplements.

## Data Availability

The datasets used and analyzed for the present study are available from the corresponding author.
